# Toward a Model for Field-Testing Patient Decision-Support Technologies: A Qualitative Field-Testing Study

**DOI:** 10.2196/jmir.9.3.e21

**Published:** 2007-07-13

**Authors:** Rhodri Evans, Glyn Elwyn, Adrian Edwards, Eila Watson, Joan Austoker, Richard Grol

**Affiliations:** ^4^Centre for Quality of Care Research (WOK)Radboud UniversityNijmegenNetherlands; ^3^Cancer Research UK Primary Care Education Research GroupDivision of Public Health and Primary Health CareUniversity of OxfordOxfordUK; ^2^School of Health and Social CareOxford Brookes UniversityOxfordUK; ^1^Department of Primary Care and Public HealthCardiff UniversityWalesUK

**Keywords:** Field-testing, patient decision-support technologies, prostate-specific antigen (PSA), prostatic neoplasms, informed choice, decision support techniques, patient education, patient participation, consumer health informatics, Internet

## Abstract

**Background:**

Field-testing is a quality assurance criterion in the development of patient decision-support technologies (PDSTs), as identified in the consensus statement of the International Patient Decision Aids Standards Collaboration. We incorporated field-testing into the development of a Web-based, prostate-specific antigen PDST called Prosdex, which was commissioned as part of the UK Prostate Cancer Risk Management Programme.

**Objectives:**

The aim of this study was to develop a model for the future field-testing of PDSTs, based on the field-testing of Prosdex. Our objectives were (1) to explore the reactions of men to evolving prototypes of Prosdex, (2) to assess the effect of these responses on the development process, and (3) to develop a model for field-testing PDSTs based on the responses and their effect on the development process.

**Methods:**

Semistructured interviews were conducted with the men after they had viewed evolving prototypes of Prosdex in their homes. The men were grouped according to the prototype viewed. Men between 40 and 75 years of age were recruited from two family practices in different parts of Wales, United Kingdom. In the interviews, the men were asked for their views on Prosdex, both as a whole and in relation to specific sections such as the introduction and video clips. Comments and technical issues that arose during the viewings were noted and fed back to the developers in order to produce subsequent prototypes.

**Results:**

A total of 27 men were interviewed, in five groups, according to the five prototypes of Prosdex that were developed*.* The two main themes from the interviews were the responses to the information provided in Prosdex and the responses to specific features of Prosdex. Within these themes, two of the most frequently encountered categories were detail of the information provided and balance between contrasting viewpoints. Criticisms were encountered, particularly with respect to navigation of the site. In addition, we found that participants made little use of the decision-making scale. The introduction of an interactive contents page to prototype 2 was the main change made to Prosdex as a result of the field-testing. Based on our findings, a model for the field-testing of PDSTs was developed, involving an exploratory field-testing stage between the planning stage and the development of the first prototype, and followed by the prototype field-testing stage, leading to the final PDST.

**Conclusions:**

In the field-testing of Prosdex, a Web-based prostate-specific antigen PDST, the responses of interviewed men were generally favorable. As a consequence of the responses, an interactive contents page was added to the site. We developed a model for the future field-testing of PDSTs, involving two stages: exploratory field-testing and prototype field-testing.

## Introduction

Field-testing is increasingly recognized as an important step in the quality assurance of patient decision-support technologies (PDSTs), interventions commonly known as decision aids. This was underlined by the International Patient Decision Aids Standards (IPDAS) Collaboration consensus statement on PDST quality, the product of a Delphi process involving all major stakeholder groups, at the end of which nine domains of PDST quality criteria were agreed upon [1]. One of these domains was systematic developmental process, which incorporated the criterion of field-testing in order to show that a decision aid was acceptable to patients [1]. IPDAS, however, did not define field-testing, and, more broadly, the PDST/decision aid literature gives very little guidance in this respect [2]. Furthermore, there are potentially two processes encapsulated in field-testing: (1) the development of a prototype with users, and (2) the “live” testing of a refined prototype.

In 2002, we were commissioned to develop a Web-based, prostate-specific antigen (PSA) PDST, called Prosdex, and included field-testing as part of the development process [3]. Prosdex formed part of the UK Prostate Cancer Risk Management Programme strategy, led by the National Cancer Screening Programmes, which had, at its heart, the concept of informed choice in PSA testing [4]. According to the strategy, UK men interested in the PSA test would be provided with information to enable them, with their family doctor, to make an informed decision. Prosdex was developed in order to present this information in the format of a Web-based, multimedia, interactive PDST. This opportunity for users to explore the information presented on PSA explains the full name of Prosdex: Prostate-Specific Antigen Decision Explorer [3].

Prosdex presents evidence-based information about prostate cancer and PSA testing, encouraging users to weigh the pros and cons of testing for themselves. Much of the information came from an earlier, paper-based decision aid for PSA testing commissioned as part of the UK Prostate Cancer Risk Management Programme and approved by its Scientific Reference Group [4]. Of particular importance in that decision aid were the statistical/epidemiological data, which allowed us to present some of the more controversial issues, such as the validity of the PSA test. For instance, we stated in Prosdex that two thirds of men with a raised PSA test do not have prostate cancer. The development of Prosdex was also underpinned by a systematic review of PSA decision aids, undertaken not only to garner information on extant PDSTs, but also to explore their effects. We found that the evaluations of PSA decision aids demonstrated, fairly consistently, an improvement in knowledge about PSA testing and prostate cancer; in contrast, however, no clear effect was found on PSA testing itself [5]. The findings were broadly similar to those of a Cochrane review of the effect of PDSTs that considered a range of clinical domains [6]. This review found that patients who use PDSTs participate more, know more, have more realistic expectations of benefits and harms, and are more likely to receive an option with outcomes they most value [6,7].

Narrative is also employed in Prosdex to present information. Specifically, there are 25 video clips of enacted patient experiences about the PSA test and subsequent investigations/treatments. The transcripts for these clips were obtained from a qualitative study of men’s experiences of PSA testing [8]. Informed choice is actively encouraged in Prosdex through structured decision support in the form of a decision-making scale. The link to this functionality lies on the top right of each page, thereby allowing users to weigh the impact of the information in that particular page on their decision-making process. Specifically, they are able to indicate whether they are for, against, or undecided about PSA testing on the basis of that information. Each decision is then added, or “stacked,” in the decision summary to produce a cumulative result for the pages viewed. Prosdex has been designed to cater to the needs of users with visual and hearing difficulties. Consequently, there is a voice-over option to which the website defaults, but which can be switched off; there are also subtitles for the video clips.

**Figure 1a:**
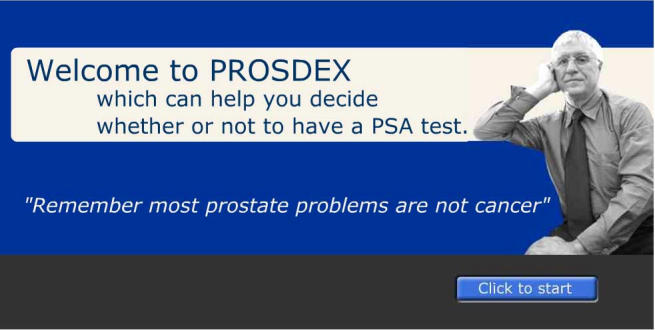
Prosdex screenshots

**Figure 1b:**
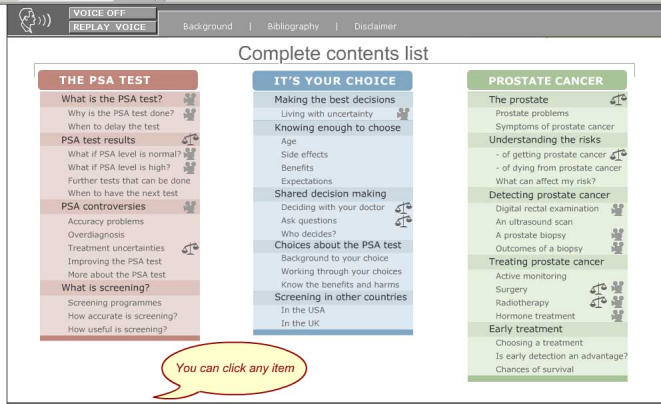
Prosdex screenshots

In this study, we attempted to capture the process of field-testing Prosdex by interviewing men who used it. Furthermore, by introducing evolving versions of Prosdex, we hoped that the series of interviews would help us, as developers, to identify strengths and weaknesses and modify the prototype. Beyond this, however, we wanted to explore the role of field-testing in the development of PDSTs. Specifically, our aim in this study was to develop a model for future field-testing of PDSTs*.* Our objectives were: (1) to explore the responses of men to evolving prototypes of Prosdex, (2) to assess the effect of these responses on the development process, and (3) to develop a model for field-testing PDSTs based on the responses and their effect on the development process.

## Methods

A qualitative study design was employed using semistructured interviews and incremental prototypes of Prosdex. Men between the ages of 40 and 75 were recruited, the target age range for the UK Prostate Cancer Risk Management Programme. The men were recruited from two family doctor practices in Wales, United Kingdom: one in a coastal/rural town and the other in a postindustrial town. The men had previously participated in a randomized controlled trial examining the effect of a brief patient decision aid—a written one-page leaflet given to the intervention group. All of the men in that trial completed a written questionnaire exploring their knowledge of and attitudes toward PSA testing and their intention to be tested [9]. At the end of the questionnaire, the men were asked to indicate whether they would be interested in participating in the qualitative study, and those who did so were sent invitation letters, information sheets, and consent forms by the research team.

Men who agreed to participate were contacted, and arrangements were made for them to view a stand-alone prototype of Prosdex on a laptop, in their homes, in the presence of one of the researchers (RE), who sat behind them. The researcher did not impart any advice or answer questions on content matters during the viewing—questions were, however, addressed during the subsequent interview. Technical questions, particularly those relating to difficulties in using Prosdex (eg, navigational problems), were answered contemporaneously. In the event of the men being unable to use a computer mouse, the researcher performed this function for them, opening specified Web pages but not giving any direction on use. The men were asked to indicate when they had finished using Prosdex and were then given a 5- to 10-minute break before the interview.

In the interview, the men were initially asked for their opinion of Prosdex in general. Then, they were asked for their views on specific aspects of Prosdex, some of which are listed in Table 1.

**Table 1:** Specific aspects of Prosdex discussed in the interview

	**Aspect of Prosdex**
1	Front page
2	Voice-over
3	Ease of use of the left-hand heading section
4	Section headings: clear or confusing
5	Decision scale and decision summary page
6	Video clips
7	**Information in Prosdex:** Presentation, and ease of use, in center panel Detail of information Legibility of information Explanation of information Format: text, video clips Satisfaction with the information Views on the presentation of different outcomes
8	Relative preference for leaflet or Prosdex
9	Suggestions for making Prosdex easier to use
10	Time taken to use Prosdex
11	Aspects that were most/least helpful

The transcribed interviews were coded independently by RE and GE with qualitative software, Atlas-ti (version 4.1), and using the technique of constant comparison [10,11]. The coded transcripts were then subjected to thematic analysis by RE and GE. Technical issues that arose during the viewings were noted by RE, and those, in addition to comments from the interviews, were fed back to the multimedia designer. Feedback occurred after a group of men had viewed each prototype, in order to maintain version control. This iterative development process resulted in evolving prototypes of Prosdex. The content of the site, however, stayed the same throughout. Finally, after analyzing the men’s responses and subsequent changes to Prosdex, we developed a model for field-testing PDSTs.

## Results

The results are presented in five sections: (1) characteristics of the interviewed men, (2) data from themes, (3) analysis of data, (4) outline of changes made to Prosdex, (5) a model for field-testing PDSTs.

### Characteristics of the Interviewed Men

A total of 27 men were interviewed after using Prosdex, between September 2004 and February 2005, and they were grouped according to the prototype viewed. There were five groups; the group that used prototype 1 (7 men) was deliberately larger than the others in order to capture the majority of the technical problems before the production of further prototypes. The men viewed Prosdex for between 15 and 45 minutes.

**Table 2:** Characteristics of the interviewed men

Prosdex Prototype	Date of Viewing	Number of Men	Age Range	Median Age	Number Who Previously Had PSA Test	Number Unable to Use Computer Mouse
1	September 2004	7	49-70	55	0	2
2	October 2004	6	50-76	60	1	1
3	November 2004	5	42-70	60	3	0
4	January 2005	5	50-70	68	1	0
5	February 2005	4	43-68	58	1	1
						
**Total**		**27**	**42-76**		**6**	**4**

### Data From the Themes

Two main themes were identified, and they are shown in Table 3 and Table 4, along with their categories and illustrative quotations. The respondent/man is identified according to the prototype group; for instance, the third man to use and be interviewed about prototype 4 is P4,i3. It should be noted, however, that the quotations are presented in relation to the themes for the whole sample, not in relation to the developing prototypes.

**Table 3:** Theme 1: Responses to the Information Provided in Prosdex - Categories and quotations

**Category**		**Quotations**	
detail		(Q) Do you think there was enough information, or too much? P1,i2: *I didn't think there was too much. I think on the question on symptoms, I don't know whether it was possible to give any more information, because once you start giving instances or factors, I suppose it's impossible to be exhaustive in any case, and therefore you can only give a broad brush.* P1,i3: *It made things a lot clearer, but I am slow on the uptake anyway. It takes a long time for things to sink in at the moment.* P1,i4: *I was taking it all in, so I think there was enough to be honest. Maybe in time I will think about something, and I should have asked this or that; it's like everything else. I think there was enough for the first time to be honest.* P1,i7: *Very informative. It raises some points which obviously concern you. The sexual activity aspect. It's very comprehensive; it spells everything out for you.* P4,i2: *I would like to know more but I'm not sure, after having looked at the website, whether the information is actually in the public domain anyway. With the test being as inaccurate as it is.*	
		(Q) Did the information go into enough detail for you? P5,i1: *Oh certainly enough detail. There was definitely enough to make a decision.*	
balance		P1,i6: *It gave you the pluses and the minuses quite well.* P3,i4: *I thought it was very informative; it told you the advantages and disadvantages, and the percentage of possibility of having the problem with the prostate, and not be detected, which you really don't want to hear that. What you want is a positive answer all the time, but obviously in life you can't have that.* P4,i1: *There perhaps ought to be more emphasis on the fact that benign prostatic hypertrophy was a perfectly normal characteristic of an aging male population, but on the other hand there is the possibility that it might be either an aggressive or an unaggressive nature, and that initially people don't need to go any further than that.*	
suggestions to improve the information		P1,i2: *The one point that I did think could be improved was where it said, "What is the practice with regard to PSA in other countries?" And it only mentioned America, and ideally, I think it should compare to other European countries.* P1,i3: *As I say, being a layman, not a lot of people know where the prostate is and all that. There could be a little bit more then.*	
		(Q) Was there any information that you would have liked, but you couldn't find? P1,i5: *The diagram was quite informative. I would have liked more detail. I would have liked more pictures as well.*	
		(Q) Was there any other information that was not there? P1,i7: *Possibly some statistics on tests that have been done, particularly as they used a comparison. They showed something about comparing the frequency of when these tests are carried out, say like in the USA, and they used a similar screening program for breast cancer for women. I think it would be interesting to see what sort of statistics have been gained.* P2,i6: *But they didn't say if you're 75-85 how it would be likely to affect you or not affect, a purely selfish point of view. Having reached 75 now I want to know what are the prospects for me over the next ten years.* P4,i3: *That phrase, “up to 1 in 5,” that phrase doesn't mean anything. And I really think that that shouldn't be there.*	
		(Q) Are there any other types of information that you would like to see? P5,i3: *A bibliography would have been useful, if there were references to more detailed information.*	

**Table 4:** Theme 2: Responses to Specific Features of Prosdex - Categories and Quotations

**Feature**	**Category**		**Quotation**
navigation			P1,i6: *I think it could have been slightly better. I think it would have been better if you had gone directly from one section to the other, if you are guided better from one section to the other in a better way. I think it was going backwards and forwards all over the place. It could be a little bit confusing, and you could actually forget or miss bits.*
			(Q) How long did it take you to feel comfortable using it? P2,i2: *Minutes. As soon as I worked out that you could take it in any order you wished. But I was quite happy to follow along with the program.* P3,i3: *I personally found it very easy, but I would think maybe someone quite a bit older who didn’t have computer skills probably would be a little overwhelmed.* P4,i2: *Very easy to navigate round, and I understood it, so I would think 90% of the population could understand it.*
video clips	balance		(Q) Do you think there were enough video clips, or too many? P1,i2: *I don't think there were too many. There were two videos where they were referring to similar symptoms about radiotherapy and diarrhea. No, I thought the balance was right.* P1,i5: *I thought they were very good actually. Some of them were a bit disheartening, but it depends on people's pain level. I mean I have got quite a high pain level.*
	detail		P2,i6: *They looked a little bit staged, like actors saying the words...just a little bit too rehearsed. And very brief, the comments were very brief. Could you condense those down into less choices but longer explanations?* P5,i2: *Well, it's enough detail to talk about it, but would be better detail to actually see it. It would give you a better idea of what you've got to do and what you've got to go through. Like the operation.*
voice-over	clarity		P1,i7: *Very clear, and an easy pace to listen to as well. It neither went too fast nor too slow.* P2,i1: *I think the thing was, that you didn't know whether to listen to the voice or read the words, and then go back and hear the voice again. I wasn't sure about that. If I had to go through the program another time, I would get to know my way around it better, let's put it that way.* P2,i5: *I found I was starting to read over it then waiting for the voice to catch up.* P3,i3: *It saves my eyesight and it also slows me down. I would probably, if I was purely reading it, I would probably speed read it and skip quite a lot more. So I found the voice very, very helpful.*
decision-making scale	limited utility		P1,i5: *On about four or five things, but generally I got too engrossed in the bit on the left reading through it all, and listening to it as well.*
			(Q) Did you use that? P1,i6: *I didn't actually, because I was going through the rest of the info, so I didn't bother. Maybe I should have done, I'm sorry.* P1,i6: *I think it may have been easier if the decision scale was at the bottom, underneath the section you are reading, as opposed to a little box on the top right. So as you go through it, click it, then go to the next page, click it on the bottom.* P2,i1: *I feel that before you moved on, if there was some sort of audio or visual prompt so that if you haven't clicked on the decision box it prompts you - maybe a little pop up or a bleep or something to tell you that you hadn't ticked the decision box.* P2,i2: *There wasn't any indication of where to use it. Whether you just had to use it at the end or at every page you'd read. I wasn't sure what to do.* P2,i3: *I didn't actually go to that, because I knew what I'd put in.* P4,i2: *I did it in my head. I'm used to making decisions, so I don't need a little Geiger counter to tell me.*

### Analysis of Data

#### Theme 1: Responses to the Information Provided in Prosdex

Three main categories were identified: (1) detail, (2) balance, (3) suggestions to improve the information.

##### Detail

In general, the men were happy with the amount of information provided (P1,i4), although there was an appreciation of the difficulty in deciding on the level of detail (P1,i2) and a realization of the weakness of the evidence base (P4,i2). Openness on sensitive issues was commended (P1,i7), and there was some evidence that the site helped to clarify some of the complexities and uncertainties of PSA testing (P1,i3). This level of detail was noted, in some cases, to be helpful for the decision-making process (P5,i1).

##### Balance

Mostly positive comments were made about the balance of the information on the site. The presentation of uncertainty was commended (P1,i6), and there was an appreciation of the difficulties involved in presenting such information (P3,i4). Nonetheless, there were some dissenting comments in this respect; for instance, one man would have preferred a greater emphasis on the benign nature of most prostate conditions (P4,i1).

##### Suggestions to Improve the Information

Specific suggestions were made to improve on the information on the site. These included a desire for more background anatomical information (P1,i3), more diagrams (P1,i5), more age-specific information (P2,i6), and a preference for a bibliography (P5,i3). In addition, there were comments on the lack of information about other European countries (P1,i2), and some criticism of the presentation of the statistical information.

#### Theme 2: Responses to Specific Features of Prosdex

The four specific features that were discussed in greatest detail were (1) navigation of the site, (2) video clips, (3) voice-over, (4) decision-making scale.

##### Navigation of the Site

The navigation difficulties with prototype 1 resulted in the most significant criticism of Prosdex (P1,i6). Men using later prototypes were less critical of the navigation, almost certainly due to the interactive contents page developed after field-testing of prototype 1 (P2,i2).

##### Video Clips

The two main categories identified here were those of balance and detail. In terms of balance, the responses were positive (eg, regarding our presentation of contrasting opinions and experiences) (P1,i2). There was also an appreciation of the difficulty in striking such a balance, particularly when dealing with sensitive issues (P1,i5).

With respect to detail, there were two specific criticisms. One man expressed a desire for more graphic detail in relation to the descriptions of prostate investigations and treatments (P5,i2). Another man expressed a preference for less choice of video clips for a particular issue, and for greater detail in those clips (P2,i6).

##### Voice-Over

The category of note here was clarity, and, in this respect, the responses were mixed.

Only one man (P2,i4) decided to switch off the voice-over using the button provided. Of those who left the voice-over on, some gave positive responses (P1,i7); in particular, one man found the process of reading to be made easier (P3,i3) with the voice-over. In terms of negative responses, one man found the voice confusing (P2,i1), and another found the voice-over to restrict his use of Prosdex(P2,i5).

##### Decision-Making Scale

The significant category here was limited utility, a consequence of the men making little use of the decision-making scale (P1,i6; P2,i3; P4,i2). The reasons given for the minimal use of the scale varied. For one man it related to the positioning of the scale on the screen (P1,i6); for another, it seemed to be caused by a limited understanding of when to use the scale (P2,i2). As the viewing of Prosdex progressed, one man focused on the content and stopped using the scale (P1,i5). One solution offered for this was audiovisual prompts/reminders to use the scale (P2,i1).

### Outline of Changes Made to Prosdex During the Field-Testing

#### Navigation

The major change made to Prosdex during the course of field-testing was to improve the navigation of the site. As previously noted, men using prototype 1 found it difficult to keep a record of which pages they had viewed (P1,i6). Consequently, for prototype 2, an interactive contents page was developed that not only indicated to the men which pages they had visited, but also allowed them to navigate directly to sections of interest. This change improved the navigation significantly for the men, and no other amendments were deemed necessary in this respect.

#### Content

The content, both text and video, remained unchanged in Prosdex since the responses regarding this were generally positive, in particular about the detail and balance of the information on the site. As highlighted above, there were some specific suggestions, and these were considered by the developers. It was decided, however, that either the information was, in fact, already present in the site, or that the requested content would have overwhelmed sections that were already very detailed. An example of this was the request for a pan-European comparison of PSA screening (P1,i2). Our decision to keep the comparison at a UK/USA level was made in order to provide the UK target audience with a relevant comparison of different practices.

#### Voice-Over

Despite the mixed responses to the voice-over functionality, it was retained in Prosdex. As previously noted, only one of the respondents (P2,i4) asked for the voice-over to be switched off, and only two of the respondents (P2,i1 and P2,i5) stated that the voice-over affected their reading of the text. Furthermore, as developers of a publicly available health information site, we were obliged to make arrangements for visually-impaired users or those with reading difficulties. Finally, we were confident that the criticisms raised could be addressed by the clearly marked option on the site to switch off the voice-over.

#### Decision-Making Scale

The decision-making scale was also retained in Prosdex despite its low usage in the field-testing. Our reason for doing so was based on the original design for Prosdex, one of the key features of which was a tool for interactive decision making. There was also no evidence that the scale interfered with other components of the site, and it was agreed that some users might find it to be of benefit.

### A Model for Field-Testing PDSTs

**Figure 2:**
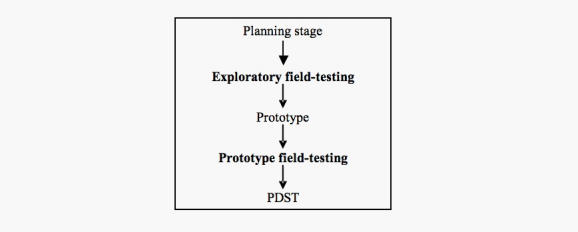
Proposed model for field-testing PDSTs

For the purposes of developing a model for field-testing PDSTs, we reflected on the qualitative data from the men’s responses and on the changes made to Prosdex*.* We found that PDST field-testing was composed of two distinct processes: (1) a process of user involvement in the development of the PDST, and (2) user trials of one or more prototypes. Consequently, for the model, we divided field-testing into two stages (Figure 2). In the first stage, which we defined as exploratory field-testing, users would be asked to look at specific components of the PDST early in its development, before the construction of the first prototype, thus allowing users to influence key decisions early on. In the second stage, which we defined as prototype field-testing, users would be shown successive prototypes, as in this study, but with reference to changes made during the development process.

## Discussion

### Summary of Main Findings

The two main themes from the interviews were the responses to the information provided in Prosdex and the responses to specific features of Prosdex. Within these themes, two of the most frequently encountered categories were detail of the information provided and balance between contrasting viewpoints. Criticisms were, however, encountered, particularly with respect to the navigation of the site. In addition, we found that the men made little use of the decision-making scale.

The introduction of an interactive contents page to prototype 2 was the main change made to Prosdex as a result of the field-testing. Other aspects of the site, notably the content, voice-over, and decision-making scale, were not changed, for two reasons. First, the collective responses did not justify radical amendments such as removing specific features. Second, there were factors other than the men’s responses to consider in the development process, notably PDST quality criteria*.* For example, one of the reasons for retaining the decision-making scale was that values clarification is an internationally recognized quality criterion for PDSTs [1].

Finally, based on our findings, a model for the field-testing of PDSTs was developed, comprising two stages: exploratory field-testing and prototype field-testing.

### Limitations of the Study

Only two family practices were used to recruit men for this study. It would have been desirable to recruit men from a greater number of practices to ensure a broader socioeconomic and geographic population distribution. Another limitation was the fact that the men had previously participated in a randomized controlled trial of a brief PSA paper decision aid in which they all had completed a written questionnaire. However, we would argue that this study differed significantly in that it focused on the details and technical aspects of a specific PDST, Prosdex, which was not featured in the trial. A qualitative methodology, semistructured interviews, was employed in this study. Arguably, however, the study design was descriptive, using qualitative techniques and employing a relatively technical, specific interview schedule, which, to an extent, is in accordance with the model stage of the complex intervention framework, as developed by Campbell et al [12].

The validity of the study’s findings is potentially open to criticism as no formal measures were employed in this respect. For instance, there was no triangulation, using data from other methods such as surveys [11]. Such an approach would, however, have been impractical in our opinion due to the dependency in this study on the presence of a researcher to facilitate the viewing of the PDST*.* Moreover, we would contend that the observational data from these viewings provided a degree of corroboration. For instance, the comments from group 1 on the navigational difficulties accorded with the researcher’s observations. Finally, respondent validation was not used as the men’s responses were dependent on their immediate recollections and views of Prosdex [11]. Corroborating these responses with the results at a later date would not, in our opinion, have been a reliable method.

### Comparisons With Previous Work

As noted earlier, we previously undertook a systematic review of evaluations of PDSTs on the topic of PSA [5]. In contrast, there are, to date, no studies that specifically consider the field-testing of PSA PDSTs. There are, however, such studies in other clinical areas, although most of these focus on the usability and acceptability of prototype PDSTs, corresponding to the prototype field-testing stage of our proposed model. For example, Irwin et al found in a pilot study that a decision aid for women with breast cancer was described as helpful by most of the users [13]. Feldman-Stewart and colleagues field-tested a PDST designed for men with early stage prostate cancer with a group of “surrogate patients”—men, without prostate cancer, of the same age as the target group of the PDST [14]. It was observed that the men were able to understand the information provided and that most were able to express treatment choices. In a noncancer setting, Lalonde et al found high levels of acceptability for a PDST aimed at improving the knowledge of patients with hypertension/hyperlipidemia [15]. Finally, and significantly, in the context of a multimedia Web-based PDST such as Prosdex, Diefenbach and Butz field-tested a multimedia interactive education system for prostate cancer patients and found high levels of acceptability [16].

The importance of prototype field-testing was highlighted by O’Donnell and colleagues in a review of the implementation of patient decision aids in clinical practice [7]. One of the significant barriers for implementation was described as “usability for diverse patients.” Specifically, the authors noted the lack of evidence on the assessment of the readability of PDSTs—a weakness shared by Prosdex—though they welcomed the finding, in the Cochrane review inventory, that most PDSTs were developed for general audiences (eg, grade 8 reading level) [6]. O’Donnell et al suggested further research on how PDSTs could improve the decision quality for people who vary by demographic characteristics. This is an important statement as it extends the potential scope of prototype field-testing. Moreover, there is a strong argument that our proposed second stage of field-testing only becomes valid if it has taken into consideration the diversity of the target audience.

There is an even greater research deficit for the exploratory field-testing of PDSTs. In one of the few studies available, Sawka et al described the development of a decision aid for choice of surgical treatment for breast cancer [17]. Notably, the study involved a needs assessment stage during which focus groups were held involving women with a previous diagnosis of breast cancer, and which considered issues such as information the women wished they had received at diagnosis. Subsequently, the decision aid was developed in conjunction with a steering group that revised various drafts of the aid. Finally, in a pilot study, almost all of the women responded positively to the decision aid. This twin approach of needs assessment and pilot study forms a strong basis for the development of a decision aid and, moreover, corresponds, in our opinion, to our proposed two-stage model for field-testing PDSTs.

The paucity of research into field-testing has implications for developers of PDST quality criteria. As previously mentioned, field-testing is, at present, regarded as an important criterion in the IPDAS framework. Moreover, this framework gives direction for the development process of PDSTs. Arguably, components of that development process are very similar to the two stages of field-testing that we propose. This is particularly true of the exploratory stage, and it again raises the question of the definition of field-testing. What is certain, however, is that with such little understanding of this criterion, it is difficult to contend, at present, that firm assessments can be made against it [1].

### Implications for Clinical Practice and Future Research

Our proposed two-stage model and, in particular, the exploratory field-testing stage, raises a number of challenges for developers of PDSTs, not least of which is the difficulty of accommodating it within the pressures of deadlines and budgets. There is also the challenge of balancing the opinions of users with those of experts/scientific reference groups, particularly in situations of clinical uncertainty/equipoise. Arguably, the model is too simplistic in that it presupposes a linear progression from exploratory to prototype field-testing. In reality, more complex PDSTs might follow a different development path wherein the factual content, for instance, would require both exploratory and prototype field-testing in order to develop other features of the PDST, for example, videos of patient experiences. Moreover, the model does not take into account contextual factors, such as the influence of family/friends and health professionals, which could have an impact on the utilization of PDSTs in a natural setting. Nevertheless, the principle of two-stage field-testing for PDSTs, whether applied in parts or as a whole, still holds true in our opinion; we suggest further research to test it and other future models of field-testing. In doing so, it is hoped that reviewers of PDSTs, and international standard groups such as IPDAS, will have at their disposal a clearer definition of field-testing.

### Conclusions

In the field-testing of Prosdex, a Web-based PSA PDST, the responses of interviewed men were generally favorable. As a consequence of the responses, an interactive contents page was added to the site. We developed a model for the future field-testing of PDSTs involving two stages: exploratory field-testing and prototype field-testing.

## Abbreviations

PSA: prostate-specific antigen
PDST: patient decision-support technology
IPDAS: International Patient Decision Aids Standards Collaboration
